# Case of Instrumental Delivery

**Published:** 1872-11

**Authors:** L. A. Harcourt


ART. II.—*Case of Instrumental Delivery. Case of Imperforate Anus.* By L. A. HARCOURT, M. D.

CASE I. In August, 1871, was called to see Mrs. M., a German, aged 37 years, married at 17, in labor with her first child. She had never before been pregnant, and she and her husband had long despaired of having any issue. For nearly seventeen long years their efforts at procreation had been fruitless, and now, when the happy hour had come—the hour that was to witness the fruition of their fondest hopes, and gladden their hearts by presenting them with an heir to their joint affections and worldly goods—nature seemed to be contravened, and wholly inadequate to the accomplishment of the task before it. Sixty hours had elapsed since the commencement of her labor, but for the last twelve, the uterus had been in a state of inertia. She had been attended for two days by some quack, or self-constituted doctor, who, having done all the mischief he could with ergot, left her in her trouble, saying nothing more could be done. The minister was there, and had prepared her for death. Such was the history of the case given by the nurse on my arrival.

When she saw me enter the room she feebly exclaimed, "too late! too late!" I emphatically answered "nein! nein!"

A vaginal examination found the head in the pelvic cavity, between the two straits, the vertix presenting, occiput to the left anterior. The head appeared to be of normal size, but the pelvis seemed to be contracted at the inferior strait, especially in the antero-posterior diameter, and the unyielding coccyx rendered it more so. The vagina was dry and hot, and the scalp corrugated. The os uteri was well dilated and soft, the anterior lip lower down than the posterior.

The woman herself was well nigh exhausted from the protracted struggle. All hope and courage gone. The pulse was feeble and fluttering, respiration labored, tongue dry and brown, lips parched, teeth black with sordees, extremities cold, and the surface covered with a clammy sweat. It was not a promising case. To my mind, there was but one indication, immediate delivery with the forceps.

I told her husband she would inevitably die if left to the resources of nature, while a speedy delivery might possibly save her life, and that of her child, if it were still living, which was somewhat doubtful. He consented, and I gave the patient some brandy before commencing the operation. I then applied the forceps, rotated the head to the right, bringing the occiput under the pubis and in ten or fifteen minutes completed the delivery. There was not the slightest contraction of the uterus during the operation.

The child was asphyxiated, but after a few faithful efforts at artificial respiration, it showed unmistakable signs of life, to the great joy of those who had so long looked for its coming.

I never attended a case where I was more anxious that the child should be alive.

I then removed the placenta, and by friction and other means compelled the womb to contract.

The woman made a good recovery, and doubtless considered herself pre-eminently blest in giving birth to a son, after so many years of fruitless expectation. And this is, perhaps, the only interesting or extraordinary feature in the case.

CASE II. January 4th, 1872, was called to see an infant two days old, which, up to that time, had never had a movement of the bowels. The midwife had gone through the whole round of infantile cathartics, before discovering that the anus was imperforate. In this condition of affairs, my services were called into requisition. I had never before seen a case of the kind, yet knew well what it was, and what I should attempt to do. The operation, however, proved far more extensive than I anticipated. Where the anus ought to be, there were three cicatrices, one passing directly through the centre from before backwards, and one on either side, in curved lines, forming an eliptical figure, perhaps one-half inch long and one-quarter wide. The integument was thick and tough.

I made an incision along the central cicatrix, its whole length, through the integument and superficial facia. This brought me to a deep layer of adipose tissue, which on being divided, puffed out like huge oil globules. Under this was some muscular tissue, which I carefully divided, and still did not find the rectum. The tissue again seemed to be adipose, which made me fear I was not cutting in the right direction. Inserting my little finger into the wound, and pushing the tissues aside with its point, I distinctly felt the closed end of the rectum. It was so high that my finger passed up to the second joint. a full inch and a half, before reaching it. It was not fixed, but would move upwards on pressure being made from below. There was a seam or cicatrix similar to the one on the outside, extending from before backwards, and showing where the parts had grown together. I held it down with a tenaculum, and made an opening into it in the direction of the seam, when there was an immediate, free and full evacuation of the bowels. With a single suture, I fastened each side of the gut to the corresponding side of the muscular tissue, and then inserted a short tube, carefully smeared with carbolic acid ointment. For a few days I dressed the wound, until the child's father, who was very poor, thought he could do it himself. A week after, he told me the child was doing well, that its bowels moved regularly, and that the wound was healing kindly. Fourteen days after the operation, he came for me, saying the child was very sick. I went to see it, and found it dying from pulmonary congestion. The bowels were soft and in good condition, nor was there the slightest trace of inflammatory action about them or the wound. The latter was almost healed, and the rectum had come down within half an inch of the external opening. I was satisfied, and so were its parents, that the operation had nothing to do with the child's death.

